# Dissecting the effect of sphingolipid metabolism gene in progression and microenvironment of osteosarcoma to develop a prognostic signature

**DOI:** 10.3389/fendo.2022.1030655

**Published:** 2022-10-14

**Authors:** Yujian Zhong, Yubiao Zhang, Sixing Wei, Junwen Chen, Changheng Zhong, Wenxiang Cai, Wenyi Jin, Hao Peng

**Affiliations:** ^1^ Department of Orthopedics, Renmin Hospital of Wuhan University, Wuhan, China; ^2^ Department of Biomedical Sciences, College of Veterinary Medicine and Life Sciences, City University of Hong Kong, Kowloon Tong, Hong Kong SAR, China

**Keywords:** Osteosarcoma, sphingolipid metabolism, tumor immune microenvironment, prognostic model, individualized therapy

## Abstract

Sphingolipid metabolism (SM) fuels tumorigenesis and the malignant progression of osteosarcoma (OS), which leads to an unfavorable prognosis. Elucidating the molecular mechanisms underlying SM in osteosarcoma and developing a SM-based prognostic signature could be beneficial in the clinical setting. This study included 88 frozen OS samples to recognize the vital SM-relevant genes in the development of OS utilizing univariate Cox regression. The Least Absolute Shrinkage and Selection Operator (LASSO) regression analysis was conducted on the SM- relevant genes to minimize the risk of overfitting. The prognostic signature was generate utilizing the multivariable Cox regression analysis and was verified in the validation cohort. Moreover, cellular and molecular mechanisms associated with SM have an unfavorable prognosis for OS patients and have been widely studied. Resultantly, an SM-based prognostic risk model was established according to critical prognostic genes (CBS*, GLB1*, and *HACD1)*, which had an excellent ability to predict the prognosis of OS patients (AUC for the train cohort was 0.887 and AUC for validation cohort was 0.737). The high-risk OS patients identified based on this prognostic signature had significantly poor immune microenvironment, indicated by significantly low immune score (mean=216.290 ± 662.463), reduced infiltrations of 25 immune cells, including NK cells (LogFC= -0.3597), CD8+T cells ((LogFC=-0.2346), Cytolytic activity ((LogFC=-0.1998), etc. The immunosuppressive microenvironment could be due to dysregulated SM of glycolipids. Further, a nomogram was constructed by integrating the SM-based prognostic signature and clinical paraments to facilitate clinical application. The nomogram could accurately predict the prognosis of OS invalids. Collectively, this study clarified the function of SM in the development of OS and helped develop a tool for risk stratification based on SM-related genes with application in clinical settings. The results of our study will aid in identifying high-risk patients and provide individualized treatments.

## Introduction

Osteosarcoma (OS) is one of the most prevalent primary malignant sarcomas of the bone in teens (1). OS has a poor prognosis and is highly metastatic. Currently, osteosarcoma therapeutics include surgical excision and neoadjuvant chemotherapy, which has enhanced the five-year survival rate in 60-70% of patients with an early-stage OS (2). Patients with metastatic or recurring OS who cannot be treated by surgery have an awful prognosis, and the five-year survival rate is lower than 20% (3). This prognosis and therapeutics of OS have been stagnant and unsatisfactory for the past 35 years. The etiology of poor prognosis of OS patients is attributed to high tumor heterogeneity due to genetic instability (4). Hence, there is an urgent requirement to recognize biomarkers for risk assessment and prognosis and to develop personalized, targeted treatment regimens.

Sphingolipids are bioactive molecules that regulate cancer cell signaling and affect tumor suppression or survival (5). Alterations in sphingolipid metabolism (SM) is involved in tumorigenesis and can serve as a candidate for therapy (6). Bioactive sphingolipid metabolites could serve as biomarkers for cancer diagnosis, disease progression, and therapeutics (7). Sphingolipids like ceramides, and sphingosine-1-phosphate (S1P), play an important role in regulating tumor cell death (8). There has been considerable advancement in our understanding of SM-associated signaling pathways and their application in cancer therapeutics. Recent studies have demonstrated the role of sphingolipids and their downstream targets in tumor development and response to chemotherapy, radiotherapy, and/or immunotherapy using novel molecular, genetic, and pharmacological techniques (5). Various studies have examined the role of SM in various cancers. Further, SM has been the focus of cancer treatment. However, the capabilities of SM-relevant genes in OS as always barely understood. Therefore, in our study, we have conducted a prognostic risk model to evaluate the prognostic value of these genes. These genes were relevant with the tumor microenvironment, infiltration of immune cells, and sphingolipid metabolism.

The tumor immune microenvironment (TIME) represents the landscape of the tumor microenvironment about immune infiltration and is crucial for the development and progression of cancers (9). Immune cells play a vital role in cellular reprogramming and alter the tumor microenvironment by secreting several molecules, which allows the neighboring cells to determine tumor survival and growth (10). Immune cells invading the tumor are predominantly the non-tumor component of the tumor microenvironment, which acts a vital role in predicting the prognosis of OS patients (11). Thus, TIME acts an essential role in the initiation and advance of the tumor. Mounting evidence suggests a close association between TIME and the pathophysiology of OS (12). Evaluation of the stage of OS aids in determining the immune status of TIME, which will help develop immune therapy and bring a better prognosis to OS patients.

In this research, we examined the impact of SM-relevant genes on the progression and survival of OS patients by conducting a comprehensive analysis of SM-related genes. In addition, we developed a risk-based scoring model to identify the prognostic relevance of genes associated with SM in OS. Our results may help develop new strategies for understanding the molecular mechanism underlying OS. This will aid in developing targeted therapeutics for OS and promote personalized OS patient care.

## Materials

### Data processing

RNA seq data of 396 skeletal muscle tissue, which served as normal samples, were retrieved from the GTEX database. RNA sequencing data (RNA-Seq) and the clinical parameters of 88 OS invalids were retrieved from the TARGET database. We exhibit the value of the two data cohorts using the FPKM formation and then log(x+1) transformed. Then we use the same gene annotation version (Human genes annotations released by Ensemble database, version GRch38.p13) to transform the ensemble ID to gene symbol. After that, we merge the two data cohort using the R package “limma” to normalize the data to remove the batch effect. Clinical information of the tumor samples included survival status, age, gender, and metastasis.

### Acquisition of genes related to SM

SM-relevant genes were gained from the GeneCards database (https://www.genecards.org/). The screening criteria for genes were the relevance score of > 4 for associated genes. The relevance score of the gene represent the relevance between the key words and the gene, which means a more significant relevance with a higher relevance score.

### Differential analysis

The linear models for microarray data algorithm (“limma” package) in the R was utilized to evaluate the differently expressed SM-relevant genes between normal samples and OS patients. Differentially expressed genes (DEGs) were screened utilizing the Wilcoxon rank sum test with the following screening criteria: | LogFC > 1 and q-value< 0.05, which was a adjusted P-value by the false discovery rate (FDR) method and reduces the rate of false positives in the final analysis results.

### Prognostic model construction

The OS invalids were randomly sorted into a train and a validation cohort, each containing 50% of the OS patients. Univariate Cox regression was utilizing to recognize genes relevant with OS prognosis using the genes expressed differently related to sphingolipid metabolism in the train cohort. LASSO Cox regression was conducted to examine the genes associated with prognosis for developing a predictive risk score model for anticipating the overall survival in OS patients using the R package “glmnet”. A tenfold cross-verification was utilized to evaluate the penalty parameter of the model. The following methodology was used to calculate the Riskscore for each patient:


$$Risk\;score=\sum_{i=1}^{n}\beta_{i}\times E_{i}$$

where n represents the total number of selected genes included in the prognostic signature, ***β*** *_i_* represents the regression coefficient of gene i, and ***E*** *_i_* represents the expression of gene i.

The “Gene Expression” column displays the expression value of genes that belong to the predictive Riskscore model. The median value of risk scores was utilized to sort all patients into low-OS and high-OS risk groups. The Kaplan-Meier survival analysis and the log-rank test were utilized to compare the overall survival of low-OS and high-OS risk groups. We conducted a time-dependent receiver operating characteristic (ROC) curve utilizing the “survivalROC” R package. The ROC curve evaluated the predictive accuracy of the prognostic risk score model. Finally, we assessed the application and reliability of the prognostic risks core model utilizing the validation cohort.

### KEGG and GO enrichment analysis

The “limma” R package was utilized to calculate the differently expressed SM-related genes among high-OS with low-OS patients. DEGs were selected by the Wilcoxon rank sum test, and the screening criteria were | LogFC > 1 and q-value< 0.05, which reduces the rate of false positives in the final analysis. The “clusterProfiler” R package was used for performing GO and KEGG pathway enrichment analysis on differentially expressed genes to recognize the significantly enriched biological characteristics and cellular function pathways. We visualized the enrichment analysis results utilizing the “enrichplot” and “ggplot2” R package.

### Independent prognostic analysis of risk scores and clinical characteristics

The risk score of each OS invalid was combined with the relevant clinical factors using the sample ID. The “limma” algorithm R package was utilized to analyze the link between risk scores and clinical parameters, including patient’s gender, age, and metastasis. Univariate and multivariate Cox regression were conducted to assess the effects of some clinical characteristics on prognosis.

### Gene Set Variation Analyses (GSVA)

We analyzed the variation level of biological processes between low-OS and high-OS groups utilizing the “GSVA” package in R. GSVA is a non-parametric and unsupervised method for assessing the alterations in the biological pathways and processes utilizing the gene expression values. The standard gene sets were constructed using the “c2.cp.kegg.v7.1.symbols” and “c5.go.v7.5.1.symbols” gene sets from the Molecular signatures database (https://www.gsea-msigdb.Org/gsea/msigdb).

### Gene Set Enrichment Analyses (GSEA)

To gain a deeper understanding of the potential mechanisms associated with SM in OS, the samples were sorted into high-OS and low-OS groups based on the risk scores. Further, the GSEA analysis was performed to determine if the differently expressed genes in two groups were significantly enriched in any biological processes or pathways. The standard gene sets were “c2.cp.kegg.v7.5.1.symbols.gmt”.

### Immune infiltration analysis

The Single Sample Gene Set Enrichment Analysis (ssGSEA) was utilized to work out the enrichment scores based on the relative frequency of expression of immune genes in each patient by comparing the enrichment score of OS samples in low-OS and high-OS groups. The stromal score, tumor purity, immune score, and estimation score were calculated for patients in the low-OS and high-OS groups. We also evaluate the quantity of eight immune cell types in tumor tissue utilizing the Microenvironment Cell Populations-counter (MCPcounter) algorithm. Moreover, the correlation between prognosis-related genes and immune function was studied.

### Protein-Protein Interaction (PPI) network

The Search Tool for the Retrieval of Interacting Genes/Proteins (STRING) web-based database (string-interaction.org) was used to conduct the PPI networks of different genes in high-OS and low-OS patients.

### A nomogram conducted for predicting overall survival

The “rms” R package was used to establish a nomogram for anticipating the overall survival of OS invalids. The nomogram was also used for age, gender, metastasis, and risk score prediction. The time-dependent calibration curves were used to estimate the accuracy of the nomogram.

### Cell line culture

Human osteosarcoma cell lines (HOS, 143B) were purchased from Wuhan Procell Life Science and Technology Co.,Ltd. (Wuhan, China). We use MEM medium contains 10% FPS and 1% Penicillin-Streptomycin to culture cell lines. The cell lines were incubated at 37°C in 5% CO2.

### Gene transfection

The GLB1 expression plasmids (CAT#: RC216106) were purchased from OriGene Technologies (Wuxi, China). We cultured OS cells in the 6-well plate for the following experiment. For the plasmid transfections, the cells were transfected using Lipofectamine 2000 according to the manufacturer’s instructions. The cells transfected by GLB1 expression plasmids were used to perform qRT-PCR, CCK-8 assay, wound healing assay and transwell assay.

### qRT-PCR

The total RNA of osteosarcoma cells was extracted by TRIzol reagent, and reverse transcription was performed with a TransScript First-Stand cDNA Synthesis Kit (TaKaRa, Japan). qRT-PCR was carried out on an ABI-7900HT Real-Time PCR System using SYBR Green MasterMix (TaKaRa, Japan). The following primer sequences were used: GLB1-F: GGCCCACAACTCATCCAACT; GLB1-R:TAATCCAGACCTGGCCCTTG. GAPDH-F: GAGTCCACTGGCGTCTTCA; and GAPDH-R: GGGGTGCTAAGCAGTTGGT.

### CCK-8 assay

The cell lines were first cultured in a 96-well plate, and CCK-8 reagents were added, which was incubated at 37°C for another 2h. The cell lines’ viability was detected by OD 450nm with an automatic microplate reader.

### Wound healing assay

The HOS and 143B cell lines were separately cultured in a 6-well plate and half of the 6-well plate were treated by GLB1 expression plasmids. Once the cell spread over the 6-well plate, use a micropipette tip to wound the monolayer and replace the total MEM medium with serum-medium. Photographs were taken at 0h, 24h, 48h after wounding under a microscope (Olympus, Japan) to verify cell migration. We use ImageJ software to calculate the area of the wound of cells. The migrate ability was evaluated by the wound healing percentage at 24h and 48h, which was calculated by the following methodology:


Wound healing percentage = (area of 0h - area of 24h or 48h)/(area of 0h) × 100

### Transwell migration assay

After a series of treatments, 4×104 cells in serum-free medium were plated in the upper chambers of a Transwell apparatus with Matrigel. Medium in the bottom chambers containing 10% FBS served as an attractant. After 24h of incubation, cells that passed through the chamber membrane were fixed with pre-cooled formaldehyde and stained with crystal violet. The cells were counted and photographed under a microscope (Olympus, Japan).

### Statistical analysis

The Wilcoxon rank-sum test was utilized to compare two groups. The Kruskal-Wallis test was utilized for comparing at least three groups. The Kaplan-Meier survival analysis was utilized to analyze the differences in survival among patients in low-OS with high-OS risk groups. Multivariate Cox regression was used to recognize the independent predictors of overall survival in OS. ROC curves were used to assess the predictive ability of the prognostic risk score model. We conducted the statistical analysis using the R package version 4.0.0. P<0.05 were considered statistically significant in all analysis. “*” represented “*p*<0.05”, “**” represented “*p*<0.01”, and “***” represented “*p*<0.001”.

## Results

### Differential analysis of SM genes in the normal samples and OS patients

**Figure 1:**
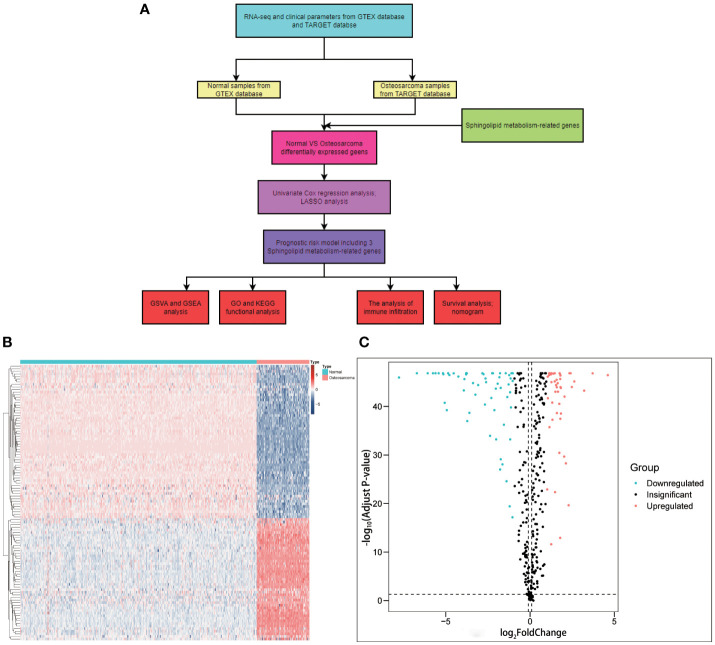
Graphical abstract and the comparison between osteosarcoma with non-tumorous tissue samples. **(A)** Flow chart of this study. **(B)** A heatmap conducted using the 106 differently expressed genes relevant with sphingolipid metabolism. **(C)** A volcano plot conducted using the 106 differently expressed genes relevant with sphingolipid metabolism. A total of 59 genes were downregulated, and 47 were upregulated in OS patients.

The article’s flow chart is given in **Figure 1A**. Differential gene analysis was performed using the screening criteria | LogFC | > 1, and q-value< 0.05. The results revealed that 59 genes were downregulated, and 47 were upregulated in OS patients. These genes were used for subsequent prognostic analysis. (**Figures 1B, C**). The encouraging results shows that SM-relevant genes expressed significantly different in OS invalids, which means we could carry out deeper investigation to comprehensively understand the role of SM in osteosarcoma.

### A prognostic risk score model conducted using the train cohort

**Figure 2:**
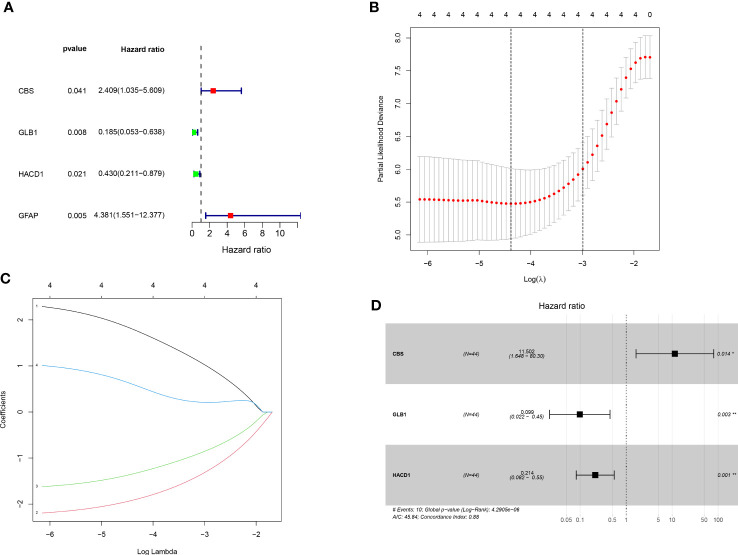
Establishment of prognostic risk assessment model. **(A)** A forest plot conducted using 17 SM-related genes associated with prognosis of OS patients. There were four SM-relevant genes associated with the prognosis of OS invalids. **(B)** LASSO coefficients for the four genes related to sphingolipid metabolism. **(C)** Gene discovery to conduct a predictive risk score model. **(D)** Forest plot of three genes (CBS, GLB1, HACD1) in the prognostic model. The CBS is a high risk gene in OS invalids, while the GLB1 and HACD1 are low risk genes in OS invalids. “*” represented “p<0.05”, “**” represented “p<0.01”.

Univariate cox regression analysis revealed four genes linked with prognosis were identified out of 106 SM-related genes (**Figure 2A**). The gene numbers were subsequently decreased using Cox regression analysis (**Figures 2B, C**). LASSO regression analysis revealed three genes (*CBS: cystathionine beta-synthase, GLB1: galactosidase beta 1*, and *HACD1: 3-hydroxyacyl-CoA dehydratase 1*) were involved in prognosis (p=0.014,0.003,0.001), and derived from these three genes, a predictive risk score model was developed (**Figure 2D**). The risk score of each sample was calculated as follows: Risk score = (2.442496727866*CBS + -2.316478283313*GLB1 + -1.54381957695251*HACD). The risk score model was utilized to sort OS invalids into low-OS and high-OS groups and then we could conduct analysis based on the different groups.

### The relevance between the risk score and clinical parameters

**Figure 3:**
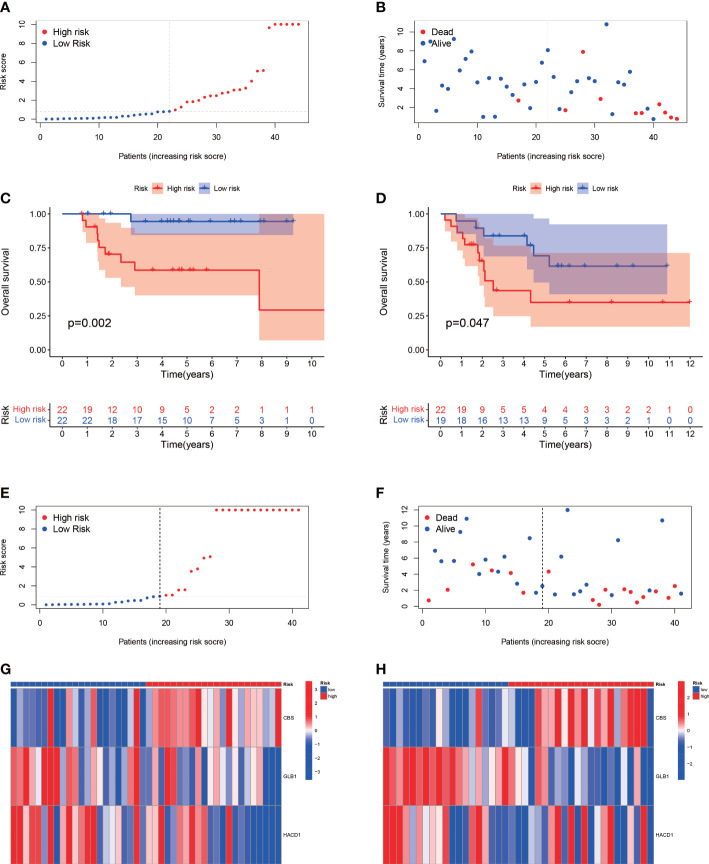
The estimation efficacy of the SM-relevant risk model in predicting the overall survival of OS patients. **(A, B)** Risk scores and distribution of OS patients in train cohort. **(C, D)** Comparison of the overall survival in the train and validation cohort between the two groups. The results show a significant different overall survival in high-OS and low-OS groups. **(E, F)** Risk scores and distribution of OS patients in validation cohort. **(G, H)** Heat map of prognostic model genes in the train and validation cohorts.

**Figure 4:**
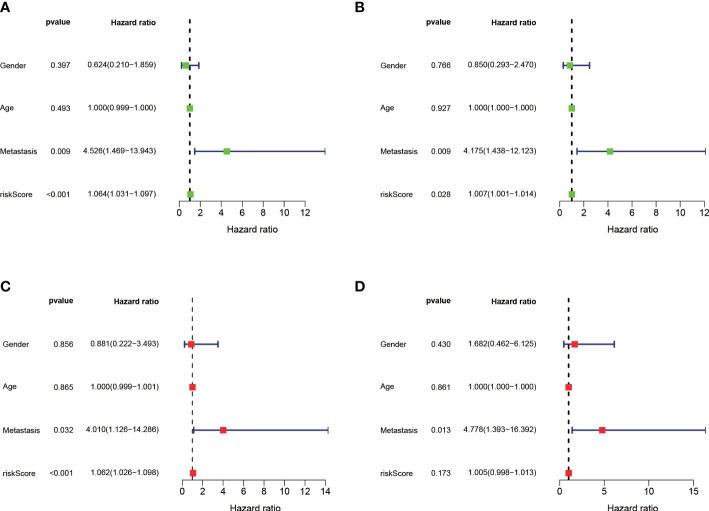
Independent prognostic analysis of the train and validation cohorts. **(A)** A forest plot conducted using univariate cox regression in the train cohort. **(B)** A forest plot conducted using univariate cox regression in the validation cohort. **(C)** A forest plot conducted using multivariate cox regression in the train cohort. **(D)** A forest plot conducted using Multivariate cox regression in the validation cohort.

**Figure 5:**
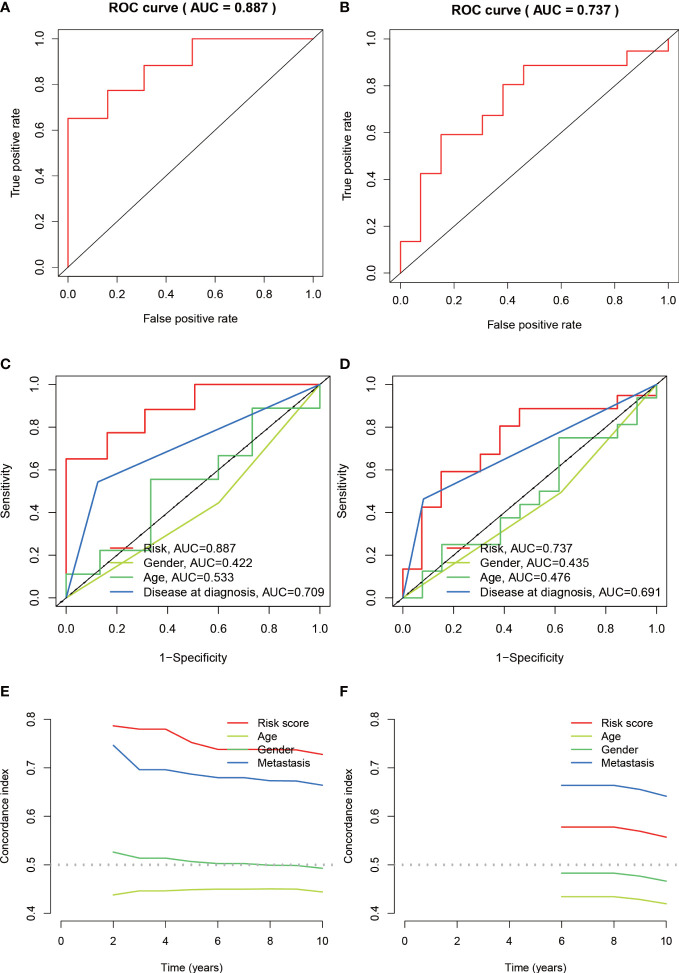
Assessing the accuracy of risk prognostic models. **(A, B)** The AUCs of 5-year survival ROC in the train and validation cohorts were 0.887 and 0.737. **(C, D)** The 5-year ROC curve combined with clinical characteristics reflects the better predictive value of risk score. **(E, F)** C-index index curves for the train and validation cohorts.

The median value of the risk score in the train cohort was used as the cutoff threshold value. Based on this threshold value and the risk scores, the patients in train cohort were sorted into low-OS (n = 22) and high-OS (n = 22) groups (**Figures 3A, B**). Patients in the high-OS group had a poor prognosis (**Figures 3B, C**). To validate the prognostic risk score model, the invalids in the validation cohort were sorted into low-OS (n = 19) and high-OS (n = 22) risk groups according to the threshold value of the train cohort (**Figures 3E, F**). A worse prognosis of high-OS patients in validation cohort suggested that the prognostic risk score model could accurately predict overall survival in OS patients (**Figures 3D, F**). The heat maps conducted to exhibit the expression of three genes (CBS, GLB1, HACD1) in OS samples revealed that CBS is upregulated in high-OS group, while GLB1 and HACD1 are downregulated in high-OS group, which is consistent with the risk models. (**Figures 3G, H**). Univariate analysis revealed that risk score, and metastasis were associated with overall survival (**Figures 4A, B**). The multivariate regression analysis shows that the risk score and metastasis were identified as independent predictors of overall survival in OS (**Figures 4C, D**). To validate the precision of the prognostic risk score model, a time-dependent ROC curve was constructed at 5- years (**Figures 5A, B**). The AUC suggested that the risk score more accurately predicts the overall survival compared to other parameters (AUC for the train cohort was 0.887 and AUC for the validation cohort was 0.737) (**Figures 5C, D**). C-index curves for risk score, gender, age, and the presence of metastasis indicated that risk score was the most accurate predictor of patient prognosis (**Figures 5E, F**). The aforementioned outcomes showed that we conduct a reliable and significant risk model to predict the prognosis of the osteosarcoma invalids.

### A nomogram constructed to predict overall survival

**Figure 6:**
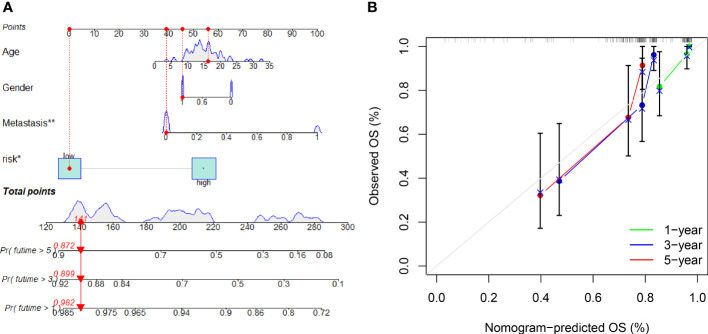
A nomogram conducted to predict the overall survival of OS patients. **(A)** Nomogram for estimating overall survival in OS patients. **(B)** The calibration plots of the nomogram. The x-axis shows expected survival, whereas the y-axis shows the actual survival. The result shows that our nomogram could estimate the overall survival acutely and reliably. “*” represented “p<0.05”, “**” represented “p<0.01”.

A nomogram with integrated age, gender, metastasis and a predictive risk score model was constructed to predict the overall survival of OS invalids (**Figure 6A**). 1-, 3-, and 5-year calibration curves show that the nomogram could precisely predict the overall survival of OS invalids (**Figure 6B**). Multiple results indicated that our nomogram is a great clinical tool to estimate the risk and prognosis of OS invalids.

### DEG and PPI network in two risk groups

**Figure 7:**
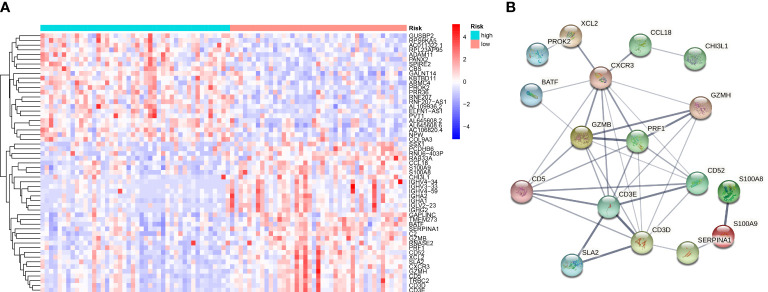
Gene Differential Analysis and PPI network for the two groups. **(A)** A heat map conducted using differently expressed genes in the two groups. **(B)** PPI network of those genes in the two groups.

Consistent with the above approach to recognize differently expressed genes in the normal and tumor groups, we identified 55 differentially expressed genes in the high-OS and low-OS groups (**Figure 7A**). The expression patterns of DEGs in low-OS and high-OS groups were analyzed using the STRING database. *CXCR3, GZMB, PRF1, CD5, GZMH, CD3E, CD52*, and *CD3D* were recognized as the hub genes, with the largest interaction network among differentially expressed proteins (**Figure 7B**). All these genes are relevant with immune function and immune diseases, which indicates that dysfunction of immune system deeply influence the risk and prognosis in OS invalids.

### Functional analysis of two risk groups

**Figure 8:**
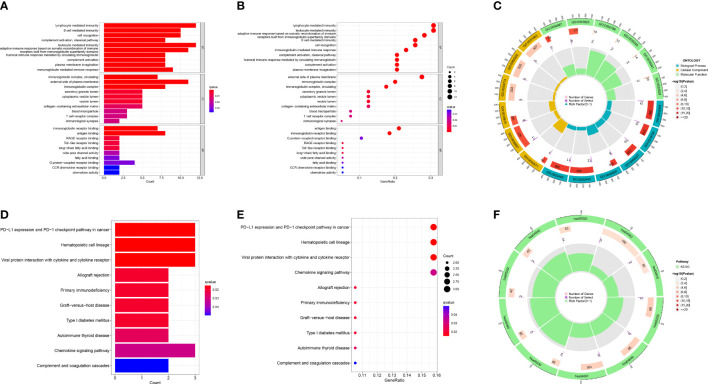
GO and KEGG functional analysis. **(A–C)** The GO enrichment analysis performed utilizing the differently expressed genes between the two risk groups. **(D–F)** The KEGG enrichment analysis performed utilizing the differently expressed genes between the two risk groups. We found multiple immune-relevant pathways were enriched such as lymphocyte mediated immunity, PD-L1 expression and PD-1 checkpoint pathway in cancer, etc.

Based on the differently expressed genes of the two risk groups, we performed functional analysis includes GO and KEGG enrichment analysis. GO enrichment analysis revealed that the DEGs were mainly enriched in immune-relevant biological processes like lymphocyte mediated immunity, B cell mediated immunity, leukocyte mediated immunity and so on (**Figures 8A–C**). Similarly, KEGG enrichment analysis also recognized some signaling pathways associated with immune and relevant diseases such as PD-L1 expression and PD-1 checkpoint pathway in cancer, allograft rejection, primary immunodeficiency, etc. (**Figures 8D–F**) These results allow us to reasonably infer that immune-relevant pathways and functions are significantly different in the high-OS and low-OS groups and may be responsible for the poor prognosis of the invalids.

### Gsva

**Figure 9:**
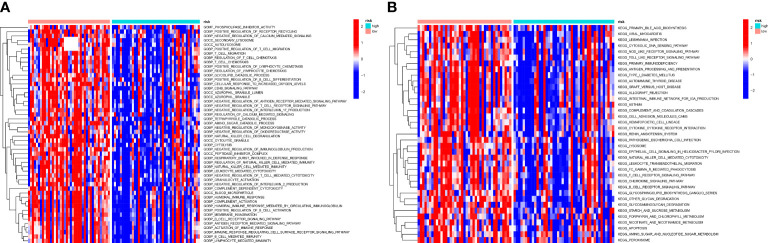
GSVA analysis of the two risk groups. **(A)** A heatmap conducted utilizing the GO gene set to estimate the variation of the two groups. There are many immune-relevant biology processes (GOBP) were downregulated in high-OS group. **(B) **A heatmap conducted utilizing the KEGG gene set to estimate the variation of the two groups, which also shows a significant change of immune-relevant pathways in high-OS group.

To understand biological processes associated with different groups, gene sets “c2.cp.kegg.v7.2” and “c5.go.v7.5.1.symbols” from MSigDB was utilized to perform GSVA. The GO and KEGG pathway enrichment analysis shows a significant enrichment of pathways such as metabolism-related pathways, immune-relevant pathways, and cellular activities in high-risk patients (**Figures 9A, B**). The differently enriched pathways between high-OS and low-OS groups included amino and sugar catabolic process, glycolipid catabolic process, regulation of T-cell chemotaxis, positive regulation of lymphocyte chemotaxis, etc. The most down-regulated pathways identified using KEGG pathway enrichment analysis were other glycan degradation, natural killer cell-mediated cytotoxicity, and glycosaminoglycan degradation. Both the two kinds of analysis revealed that immune-related biology function and pathways were significantly different. In this part, we found that glycolipid catabolic process may be relevant with the dysfunction of immune system in OS invalids.

### Gsea

**Figure 10:**
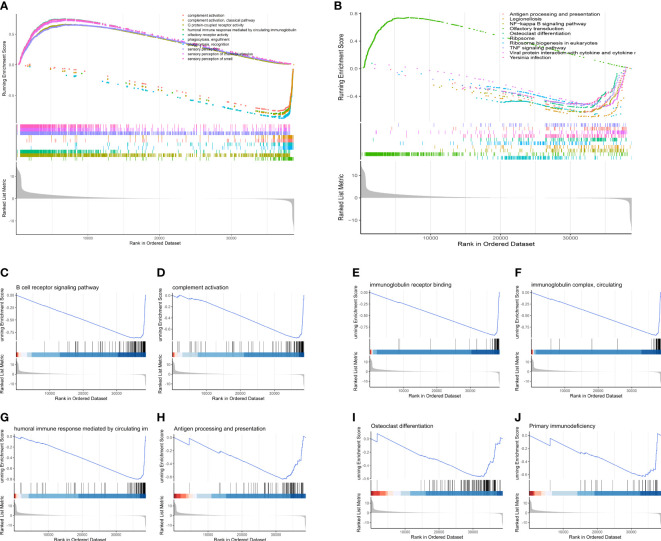
GSEA analysis of the two risk groups. **(A)** The top 10 changed pathways conducted utilizing the GO gene set. **(B)** The top 10 changed pathways conducted utilizing the KEGG gene set. Both two pictures show a downregulated immune condition, which indicates that dysfunction of immune system plays a vital role in the progression of OS patients. **(C-J)** Several immune-related and bone remodeling pathways were selected from the GSEA analysis. **(C)** B cell receptor signaling pathway. **(D)** Complement activation. **(E)** Immunoglobulin receptor binding. **(F)** Immunoglobulin complex, circulating. **(G)** Humoral immune response mediated by circulating immunoglobulin. **(H)** Antigen processing and presentation. **(I)** Osteoclast differentiation. **(J)** Primary immunodeficiency.

GSEA revealed that as compared to low-OS group, various immune-relevant signaling pathways (**Figures 10A, B**) includes B cell receptor signaling pathway (**Figure 10C**), complement activation (**Figure 10D**), immunoglobulin receptor binding (**Figure 10E**), immunoglobulin complex, circulating (**Figure 10F**), humoral immune response mediated by circulating immunoglobulin (**Figure 10G**), antigen processing and presentation (**Figure 10H**), primary immunodeficiency (**Figure 10J**) etc., were downregulated in the high-OS group. Further, with regard to bone remodeling, the pathways associated with the differentiation of osteoclasts (**Figure 10I**) were downregulated in the high-OS group. The above results help us identify an inhibited tumor immune micro environment based on groups derived from the SM-relevant genes, which proved the vital role of SM in osteosarcoma.

### Differences of the immune infiltration between the low-OS and high-OS groups

**Figure 11:**
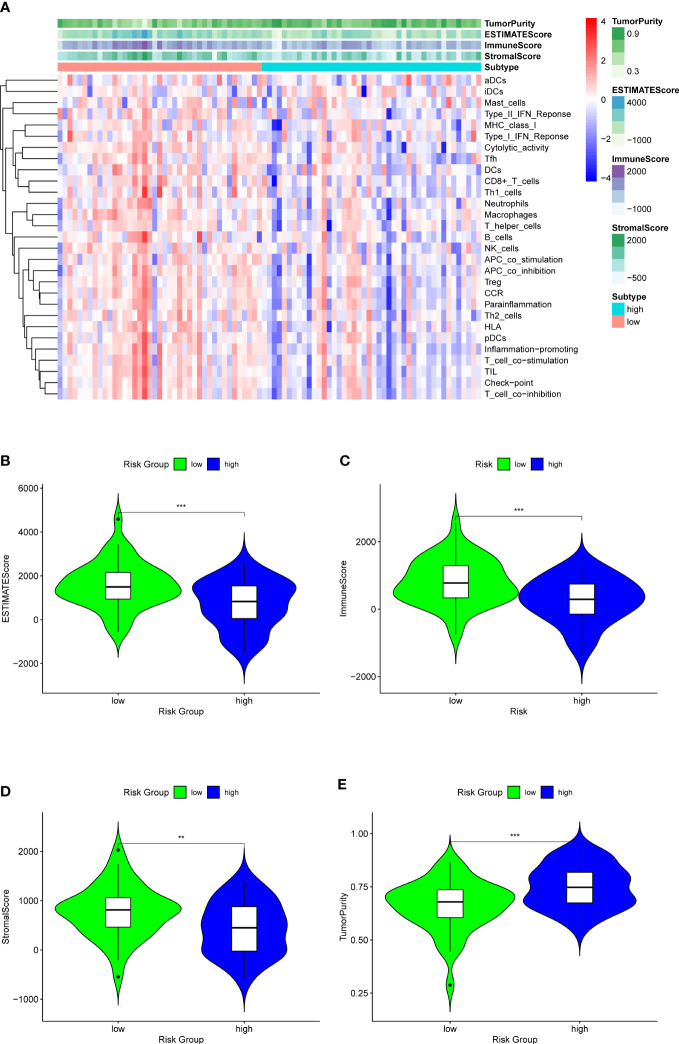
ESTIMATE analysis for the two groups to explore the immune infiltration. **(A)** A heatmap of ESTIMATE analysis of the two risk groups. **(B)** A violin plot of ESTIMATEScore in two risk groups, which shows a significant lower score in high-OS group. **(C)** A violin plot of ImmuneScore in two risk groups, which shows a significant lower score in high-OS group. **(D)** A violin plot of StromalScore in two risk groups, which shows a significant lower score in high-OS group. **(E)** A violin plot of TumorPurity in two risk groups, which shows a significant higher tumor purity in high-OS group. “**” represented “p<0.01”, “***” represented “p<0.001”.

**Figure 12:**
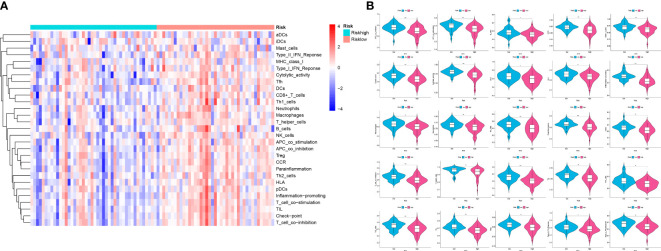
Analysis of ssGSEA in the two risk groups to explore the immune infiltration. **(A)** A heatmap of 29 immune cells and functions utilizing ssGSEA analysis in the two groups. **(B)** Violin plots of 25 significant differential immune functions between the two groups. We could know that all the 25 immune functions are downregulated, which means a immunosuppressive environment in OS patients. “*” represented “p<0.05”, “**” represented “p<0.01”,and “***” represented “p<0.001”.

**Figure 13:**
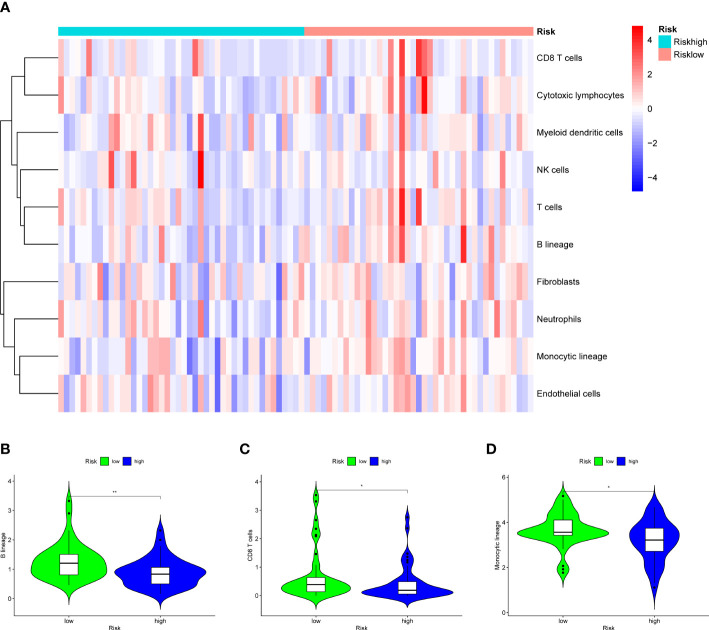
Results of MCPcounter analysis in the two groups to explore the immune infiltration. **(A)** A heatmap of 10 cell types in MCPcounter in the two groups. **(B)** A violin plot of B lineage in the two groups, which shows a significant lower level in high-OS group. **(C)** A violin plot of CD8 T cells in the two groups, which shows a significant lower level in high-OS group. **(D)** A violin plot of Monocytic lineage in the two groups, which shows a significant lower level in high-OS group. Due to above results, we could infer that SM-relevant genes may influence the immune cells to change the immune environment in OS invalids. “*” represented “p<0.05”, “**” represented “p<0.01”.

Analysis of immune infiltration was conducted to explore the distinctions in immune status among the two groups. The analysis performed utilizing the ESTIMATE algorithm revealed that samples in the low-OS group have significantly higher immunological scores, ESTIMATE scores, stromal scores, and lower tumor purity (**Figures 11B–E**). Immune infiltration was performed utilizing ssGSEA revealed that infiltration of immune cells was significantly low in the patients in the high-OS group. Totally number of 25 immune-related cells and functions were downregulated, including antigen-presenting cells (APC) co-inhibition, APC co-stimulation, B cells, chemokine receptors (CCR), CD8+ T cells, checkpoint, cytolytic activity, dendritic cells, HLA, pro-inflammatory, Type I IFN, and Type II IFN Response, etc. (**Figures 11A**, **12A, B**). MCPcounter analysis revealed that three of the ten immune cells, including B cell lineage, CD8 T cells, and monocytic lineage, were meaningfully lower in the high-OS group, whereas the other seven cells were not significantly different (**Figures 13A–D**). With these findings, we could draw a conclusion that is OS invalids, there are fewer immune cells around the tumor tissue, which may lead to a poor immune environment and promotes the migration of tumor.

### Overexpression of GLB1 inhibits the migration and invasion of human osteosarcoma cell lines

**Figure 14:**
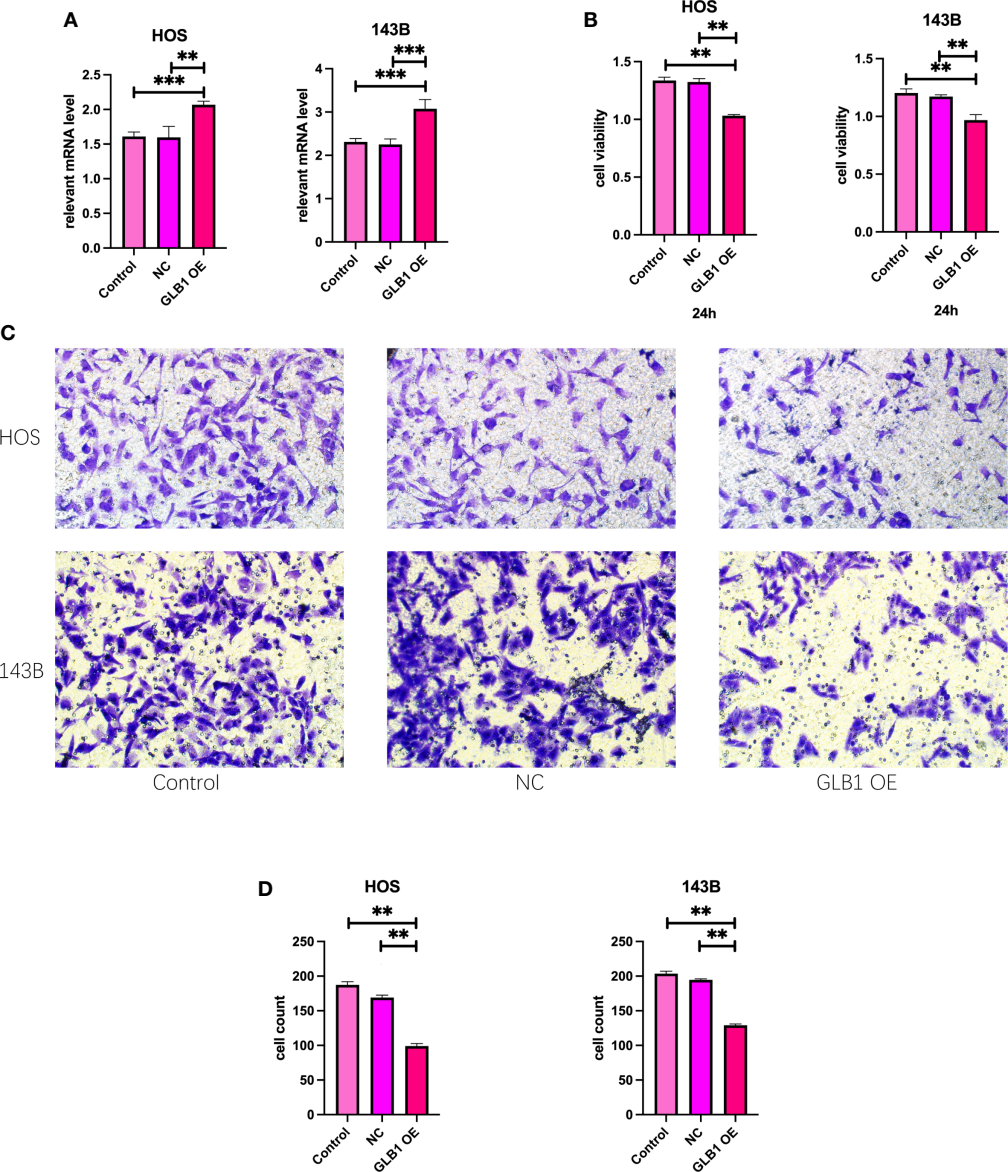
Overexpress GLB1 inhibits the proliferation and invasion of human OS cell lines. **(A)** The relevant mRNA lever of GLB1. In both the HOS and 143B cell lines, the GLB1 expression plasmids were transfected successfully. **(B)** The cell viability measured by CCK-8 assay indicates that overexpression of GLB1 inhibits the proliferation of human OS cell lines (HOS, 143B). **(C, D)** The photograph and the statistical analysis results of transwell invasion assay, which shows the overexpression of GLB1 inhibits the invasion of human OS cell lines (HOS, 143B). “**” represented “p<0.01”,“***” represented “p<0.001”.

**Figure 15:**
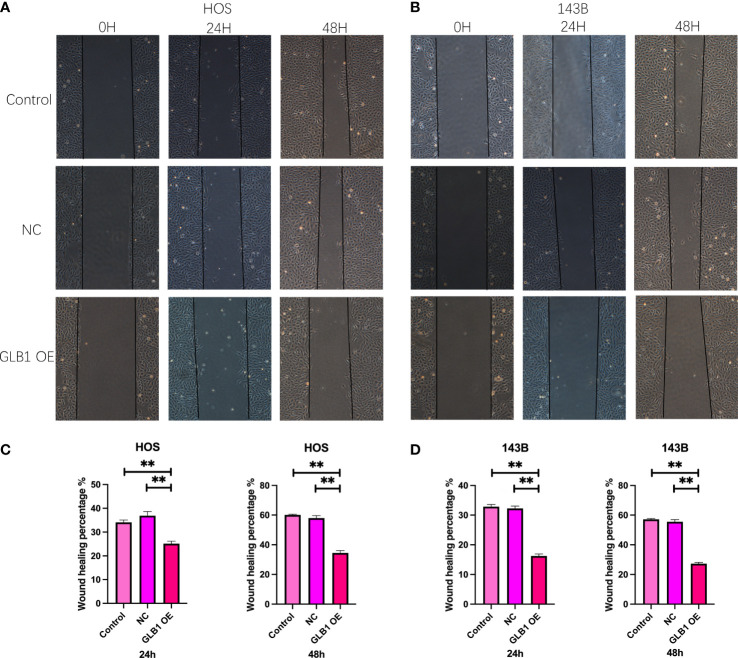
Overexpress GLB1 inhibits the migration of human OS cell lines. **(A, B)** The photograph of wound healing assay at 0h, 24h, 48h. The area is significantly smaller in GLB1 OE group. **(C, D)** The statistical analysis results of wound healing assay. All the results revealed that overexpress GLB1 could inhibits the migration of human OS cell lines (HOS, 143B). “**” represented “p<0.01”.

To confirm the results of previous bioinformatic analysis, we perform real time PCR, CCK-8 assay, wound healing assay and transwell assay. We revealed that overexpression of GLB1 could inhibits the migration of human osteosarcoma cells. According to the PCR, we overexpress the GLB1 successfully. The relevant mRNA level of GLB1 is approximately lower 40% than the control and NC groups (**Figure 14A**). Compared to the normal and NC group, the group with GLB1 expression plasmids showed significant inhibition of the cell viability (**Figure 14B**). Moreover, we found that overexpression of GLB1 inhibits the invasion of human osteosarcoma cell lines from the results of transwell assay (**Figures 14C, D**). The wound healing assay results showed that GLB1 could inhibits the migration of human cell lines (**Figures 15A-D**). The above results indicates that GLB1 inhibits proliferation, invasion and migration in human osteosarcoma cell lines (HOS, 143B), which meant our bioinformatics analysis results are reliable, and we could use our results to identify the risk of OS invalids.

## Discussion

OS is one of the most prevalent primary bone cancers prevalent in teens (1). Despite the advancement in OS therapeutics, the five-year survival probability for OS patients have remained stagnant over the past 35 years (3). Hence, there is an urgent need to create risk-based classification strategies and personalized targeted therapy regimens. In the available study, SM-related genes were identified. The analysis of immune cells revealed that patients with a poor prognosis had a poor immune profile, low immune scores, ESTIMATE scores, and a high tumor purity compared to patients with a good prognosis. Functional analysis demonstrated a relevance between the expression of different SM-related genes, immune function, and bone remodeling. Further, a prognostic risk model derived from SM-related genes could precisely predict the prognosis of OS patients. This indicates that our results could aid in developing targeted therapies for OS and assist clinicians in deciding on effective treatment regimens.

TIME plays a vital role in patient prognosis since tumor progression is directly associated with changes in the surrounding stroma and immune cells, which are an essential component of the tumor stroma (13). The ESTIMATE is a novel algorithm that predicts tumor purity derived from the expression of genes and the scale of immune cells to stromal cells within the tumor (14). The immune score worked out by the ESTIMATE algorithm quantifies the immune component of tumor tissue, whereas tumor purity refers to the number of malignant cells in tumor tissue, which is strongly associated with prognosis (15). Low immunological scores and high tumor purity have been shown to correlate with a poor prognosis. Our study used the ESTIMATE algorithm to determine the TIME for the high-OS and low-OS groups. Corresponded with previous studies, our results show that patients with a good prognosis had higher immunological scores and low tumor purity. Additionally, ssGSEA was utilized to recognize the immune status of the patients in the high-OS and low-OS groups. The results revealed that 25 of the 29 immune-relevant functions were downregulated in the high-OS group, demonstrated that samples in the high-OS group had a poor immune status, further validating the ESTIMATE and TIMER results. Therefore, it is tempting to postulate that low immunological scores and a weak immune status could be related to a poor prognosis.

Additionally, functional analyses were performed on the low-OS and high-OS groups to investigate the underlying biological mechanisms. PPI, GO, and KEGG pathway enrichment analyses were done with the differently expressed genes of the two groups. GO enrichment analysis revealed that different genes were enriched in various immunological processes. KEGG pathway enrichment analysis revealed that different genes were enriched in the PD-L1 expression and PD-1 checkpoint pathway in cancer and some immunological disorders. However, the mechanism of aberrant immune function in the high-OS patient is unclear. Therefore, GSVA and GSEA were conducted to explore the potential pathways. GSVA calculated the differences in signaling pathways in each sample derived from gene expression to evaluate alterations in the two groups (16). The following pathways differentially enriched between the two groups were regulation of T cell chemotaxis, positive regulation of lymphocyte chemotaxis, etc. The pathways significantly downregulated in KEGG analysis were other glycan degradation, natural killer cell-mediated cytotoxicity, and glycosaminoglycan degradation, among others. These results suggest that the dysregulation of the immune system could be associated with glycolipid metabolism. GSEA is a standard method to integrate gene expression matrix, which reveals the expression pattern of the genome in several conditions (17). In the current study, the GSEA results show a low immune response in the patients in the high-OS group. Together these results suggest that sphingolipid metabolism, specifically glycolipid metabolism, plays a vital role in the immune dysfunction in patients in the high-OS group.

Our aforementioned results indicate that dysregulation in SM, particularly glycolipid metabolism causes anomalies of TIME, resulting in a poor prognosis for OS patients. Recent studies have revealed that SM dysregulation could be a novel indicator of cancer and has gained considerable interest and could be a promising candidate in cancer therapeutics (18). The role of sphingolipids has been investigated in radiotherapy, drug resistance, immunotherapy, and targeted therapy, among other areas, including multiple studies investigating the function of glycolipids in malignancies (19). For instance, sphingolipids act as cell surface markers and can alter the proliferation, invasion, and migration of tumor cells (20). Further, tandem repeating sialic acid structures like those in GD3 and GD2 acts as tumor promoters, thereby contributing to tumorigenesis (21). On the contrary monosialyl gangliosides like GM1, GM3, and GM2 act as tumor suppressors. Various studies have demonstrated the molecular mechanisms by which sphingolipids influence cell signaling by interacting with molecules on the same cell membrane (cis-binding) or different cell surfaces (trans-binding) (22). Glycolipids are present on receptors of growth factors and adhesion molecules, including the integrin family, and are cis-interacting molecules. These receptors can alter cell signaling. Signaling pathways activated by receptors of growth factors and integrins, further activate downstream pathways and signaling molecules (23). The AKT, p130Cas, and paxillin enhance tumorigenesis in certain cancers like melanomas (22). Increases p130Cas phosphorylation and paxillin induce cell migration and metastasis in OS, which may be facilitated by Src family proteins (24). In addition, glycolipids play a crucial role in immune cell activities. Studies have shown that glycolipids are essential for the recruitment of immune proteins to certain membrane microstructure regions, and their association with cell surface receptors further enhances the functions of glycolipids in immunity (25). Therefore, immunological dysregulation due to sphingolipid metabolism alters the TIME in OS patients, leading to a poor prognosis.

To further study the influence of dysfunctional SM on the TIME of OS patients and its predictive significance in OS patients, a prognostic model derived from SM-relevant genes was constructed and verified in the validation cohort. In our model, all three genes identified were associated with tumor initiation and progression (26–28). *CBS* encodes a homotetrameric enzyme that catalyzes the first step in the transsulfuration pathway and converses the homocysteine to cystathionine, which is associated with various stomach, liver, and ovarian cancers and influences immune evasion (29). The protein encoded by 3-hydroxyacyl-CoA dehydratase 1 (*HACD1*) has a protein tyrosine phosphatase (PTP) catalytic domain. This PTP domain of these proteins contains a proline residue instead of the highly conserved arginine residue, which represents a different class of PTPs. Members of the PTP family act as signaling molecules regulating numerous cellular functions, which may affect the prognosis of OS patients by affecting the immune cells and biological processes (30). Galactosidase beta 1 (*GLB1*) encodes for members of the glycosyl hydrolase 35 families of proteins. Alternative splicing generates multiple transcript variants, of which one transcript may produce a preprotein that undergoes proteolysis to produce a mature lysosomal enzyme. This enzyme catalyzes the hydrolysis of a beta-linked terminal galactose residue from ganglioside substrates and other glycoconjugates (31). Studies suggest the involvement of GLB1 in prostate cancer, glioma, and other malignancies (32–34). However, the role of GLB1 in OS remains unclear. Taking together the role of GLB1 in sphingolipids, the role of GLB1 in OS should be further investigated. We hypothesize that *GLB1* triggers gangliosides to alter immune functions, thereby altering the prognosis of OS patients. Survival analysis of the train and validation cohort reveals that the constructed prognostic risk score model for OS patients could accurately predict overall survival. The results of the independent prognostic analysis show that a risk model derived from SM-relevant genes could predict the prognosis of OS regardless of the patient’s age, gender, or metastatic status. Further, the nomogram incorporating risk scores and clinical variables was constructed and calibrated, which showed similar results in predicting the survival outcomes. Taken together, these results reveal the predictive ability of SM-related genes in OS and the correlation between aberrant SM and abnormalities in TIME.

We also confirmed the role of GLB1 by biology experiment *in vitro*. The overexpression of GLB1 showed significant effects on the cell viability, proliferation, migration and invasion in the human OS cell lines (HOS, 143B). We ensured that GLB1 could inhibits the OS cells to proliferate and invasion, which means GLB1 may inhibits the growth and metastasis of OS tumor tissue in OS invalids.

There are still some limitations of the present study. Firstly, due to the lack of OS clinical samples, we use cellular experiments for validating our findings. Secondly, we have not validated how the GLB1 effects on the SM and dysfunction of immune system in OS invalids yet. The findings will be explored in our further well- designed study.

In this study, OS invalids were sorted into different risk groups derived from SM-related genes. Patients in the high-OS group behaved abnormalities in TIME, such as poor immune scores, stromal scores, glycolipids, and immunological dysfunction. These results show that aberrant sphingolipid metabolism, mainly glycolipid function, could be associated with TIME and can be a potential therapeutic target for personalized medicine.

## Conclusion

In this study, the OS invalids were sorted into high- and low-risk groups based on the association between sphingolipid metabolism and OS. Immunological and functional analyses reveal that dysregulated glycolipid metabolism impairs the activity of the immune system, resulting in a poor prognosis of OS patients. Our results offer a theoretical basis for creating novel targeted personalized therapy and aid in the risk-based classification of osteosarcoma patients.

## Data availability statement

The datasets presented in this study can be found in online repositories. The names of the repository/repositories and accession number(s) can be found below: https://xenabrowser.net/datapages.

## Author contributions

YjZ: conceptualization and visualization. YjZ and YbZ: methodology and writing—original draft preparation. YjZ, YbZ, SW, JC, and CZ: data curation. YjZ, WJ, and WC: formal analysis. HP and WJ: writing—review and editing, project administration, and funding acquisition. All authors contributed to the article and approved the submitted version.

## Funding

The present study is granted by National Natural Science Foundation of China (No. 81672154) and Hubei Provincial key research and development program, 2021BCA147.

## Conflict of interest

The authors declare that the research was conducted in the absence of any commercial or financial relationships that could be construed as a potential conflict of interest.

## Publisher’s note

All claims expressed in this article are solely those of the authors and do not necessarily represent those of their affiliated organizations, or those of the publisher, the editors and the reviewers. Any product that may be evaluated in this article, or claim that may be made by its manufacturer, is not guaranteed or endorsed by the publisher.
